# Blocking two host receptors is better than one: Insights from RSV infection in human respiratory organoids

**DOI:** 10.1016/j.omtn.2026.103071

**Published:** 2026-09-05

**Authors:** Cuncai Guo, Lifei Zhu

**Affiliations:** 1 Department of Pediatrics, School of Medicine, Washington University in St. Louis, St. Louis, MO, USA; 2 Department of Radiation Oncology, Montefiore Einstein Medical Center, 111 East 210th Street, Bronx, NY 10467, USA

## Main text

Respiratory syncytial virus (RSV) is still a leading cause of severe lower respiratory tract infections in infants, the elderly, and immunocompromised individuals.^1^ However, the host factors governing viral entry still remain poorly understood. In a recent issue of *Molecular Therapy Nucleic Acids*, Keshta and co-authors employed human induced pluripotent stem cell (iPSC)-derived respiratory organoids to ask whether four RSV fusion (F) protein-interacting host surface proteins, intercellular adhesion molecule-1 (ICAM-1), nucleolin (NCL), epidermal growth factor receptor (EGFR), and insulin-like growth factor 1 receptor (IGF1R) mediate viral entry, using CRISPR-Cas9 knockouts (KOs) and neutralizing antibodies. They found that inhibition of ICAM-1 and NCL suppressed RSV N protein expression and substantially reduced infectious viral titers.^2^ Their findings advance the mechanistic understanding of RSV entry and open a route to combinatorial host-directed therapy.

RSV is an enveloped, negative-sense single-stranded RNA virus, belonging to the *Orthopneumovirus* genus of the Pneumoviridae family. Its two major surface glycoproteins, G and F, mediate attachment and membrane fusion, respectively. F converts from a metastable prefusion (pre-F) to a stable post-fusion (post-F) state as it drives membrane merger. Pre-F is the primary target of vaccine design and monoclonal antibodies.^1^ The monoclonal antibodies nirsevimab and clesrovimab are approved for infants during RSV seasons.^1^ The vaccines Arexvy, Abrysvo, and mRESVIA are approved for adults over 60.^1^ Despite progress in RSV prevention, no curative antiviral therapy exists. A 2024 report indicates that RSV-related mortality in adults over 60 may equal or exceed that of COVID-19 and influenza.^3^

A major knowledge gap remains in identifying the host-surface receptors that bind RSV F and defining their relative contributions to entry. Several candidates have been nominated, including NCL, ICAM-1, EGFR, and IGF1R,^4^ but the supporting studies relied largely on immortalized cell lines, animal models, and primary air-liquid interface (ALI) cultures, each of which only partly reproduces the complexity and multicellular architecture of human respiratory epithelium. Human iPSC-derived respiratory organoids provide a robust 3D model that recapitulates the cellular complexity of the airway epithelium, including ciliated, goblet, club, and basal cells. They have been applied to SARS-CoV-2^5^ and, more recently, RSV. Combined with CRISPR-Cas9-mediated genome editing and pharmacological perturbation, this model enables systematic dissection of host-virus interactions.

Keshta et al. established human iPSC-derived respiratory organoids expressing four RSV F-interacting receptor candidates and confirmed their susceptibility to RSV.^2^ To further assess the functional role of each candidate, the authors generated ICAM-1 and EGFR KO organoids via CRISPR-Cas9 and blocked the function of NCL and IGF1R with neutralizing antibodies (Table 1). Of the four, only ICAM-1 and NCL perturbation clearly decreased RSV N gene expression. ICAM-1 KO organoids exhibited significantly reduced viral gene expression, alongside diminished induction of the interferon-stimulated genes ISG15 and MX1 and the proinflammatory cytokines IL-6 and IL-8, consistent with decreased viral replication rather than altered innate immune responsiveness. NCL antibody treatment produced a broadly similar phenotype. EGFR KO did not affect RSV N expression but reduced innate immune gene induction, indicating a contribution downstream of TLR3.^6^ Likewise, IGF1R inhibition had no clear effect on either viral replication or innate immune gene induction. Critically, combined ICAM-1 and NCL blockade suppressed infection more than either manipulation alone.

**Table 1:** Effect of perturbing each of the four candidate RSV entry receptors

Candidate receptor	Effect of perturbation on RSV entry (Keshta et al.)
ICAM-1 (CD54)	CRISPR-Cas9 knockout markedly reduced RSV N expression and ISG15/MX1 and IL-6/IL-8 induction
Nucleolin (NCL)	Neutralizing antibody reduced RSV N expression and infectious titer; additive with ICAM-1 loss
EGFR	Knockout did not reduce RSV N expression; innate immune gene induction reduced, consistent with a role downstream of TLR3
IGF1R	Antibody inhibition affected neither viral replication nor innate immune gene induction

ICAM-1 was reported to facilitate RSV F protein-mediated attachment or activation at the cell surface.^7^ NCL, by contrast, is thought to act as a co-receptor enhancing fusion efficiency.^8^ Placing the two factors at different steps of entry rationalizes why simultaneous blockade outperforms either single perturbation, and argues for host-directed rather than purely virus-directed intervention.

One feature of ICAM-1 worth flagging for a translational readership is that its expression is not fixed. ICAM-1 is induced in the pulmonary microvasculature by ionizing radiation in a dose- and time-dependent manner, and ICAM-1-null mice are largely protected from radiation-induced pulmonary inflammation, placing it causally upstream of lung injury rather than alongside it.^9^ If the abundance of an entry receptor is itself set by the injury state of the airway, RSV susceptibility becomes a moving target—potentially highest in exactly those patients who tolerate infection worst, such as those receiving thoracic radiotherapy or cytotoxic conditioning. Whether irradiation raises RSV entry-receptor density on airway epithelium is untested, and the organoid system described here is well suited to answering it.

A limitation is the absence of rescue experiments. Because ICAM-1 KO organoids lack ICAM-1 in every constituent cell type, re-expression is technically difficult, so an indirect effect of ICAM-1 loss on cellular composition or function cannot be excluded. NCL-neutralizing antibodies achieved only partial blockade, potentially underestimating the contribution of NCL. Inducible or cell type-specific KOs would be more definitive. The discrepancy with Griffiths et al., who used primary ALI cultures,^10^ is noteworthy and probably reflects intrinsic differences between models in cellular composition, receptor expression, and 3D architecture—itself an argument for physiologically faithful systems.

Several fundamental questions remain. Does ICAM-1 directly engage the RSV F protein or promote viral attachment by organizing membrane architecture or recruiting additional host factors? What governs recruitment of NCL to the plasma membrane during infection? The contribution of naturally occurring human genetic variation in ICAM-1, NCL, or other candidate host factors to RSV susceptibility and disease severity has yet to be established. Large-scale human genetic studies combined with functional validation in organoid models may help bridge this gap.

The therapeutic implications of these findings are substantial. Although current RSV prophylactic strategies primarily target viral proteins, this paper shows that combined inhibition of ICAM-1 and NCL markedly decreases RSV infection in a human iPSC-derived organoid model, supporting host-directed antiviral therapies. Such an approach is most valuable precisely where antibody-based prophylaxis underperforms—in profoundly lymphopenic recipients of transplant conditioning regimens and in patients on immunosuppressive cancer therapy. The platform extends to patient-derived and perturbed organoids: models from individuals with asthma or chronic obstructive pulmonary disease (COPD), or organoids given clinically relevant radiation doses, could show how epithelial remodeling—disease associated or treatment induced—alters receptor expression and viral entry. More generally, the established receptor-perturbation method is readily applicable to other respiratory RNA viruses with unresolved entry mechanisms. Systematic integration of genetic perturbation, antibody blockade, and patient-derived organoid models provides a scalable route to identifying host-directed therapeutic targets.

## Declaration of interests

The authors declare no competing interests.

## Declaration of generative AI and AI-assisted technologies in the writing process

During the preparation of this work, the author(s) used Claude (Anthropic) for language polishing and structural feedback. The author(s) reviewed and edited the output as needed and take full responsibility for the content of the published article.
